# Examining the Impact of Victimization on Girls’ Delinquency: A Study of Direct and Indirect Effects

**DOI:** 10.3390/ijerph16111873

**Published:** 2019-05-28

**Authors:** Johanna Peterson, Dana DeHart, Emily Wright

**Affiliations:** 1 Omaha Police Department, Omaha, NE 68102, USA; johanna.peterson@cityofomaha.org; 2 College of Social Work, University of South Carolina, Columbia, SC 29208, USA; 3 School of Criminology and Criminal Justice, University of Nebraska, Omaha, NE 68182, USA; emwright@unomaha.edu

**Keywords:** delinquency, girls, poly-victimization, witnessing violence

## Abstract

Previous research has acknowledged that there is a relationship between victimization and later delinquency, but the specific attributes of this relationship are unclear because measures of both direct and indirect victimization are rarely explored in a single study. We included both indirect and direct victimization to examine which form of victimization was a stronger predictor of substance use, fighting, running away, and sex work among girls committed to a juvenile justice facility. Findings indicated that direct victimization was typically a more salient predictor of delinquency than indirect forms of victimization. Further, running away and sex work appear to be unique outcomes that are particularly likely when girls experience direct rather than indirect victimization. Findings are summarized with implications for health and public policy.

## 1. Introduction

There is a general consensus in the behavioral sciences that there is a direct association between victimization and criminal or delinquent offending [1]. This is a serious public health and policy concern, as we now know youth are exposed to more violence and victimization than previously identified [2,3]. Girls and women, in particular, are at a high risk of polyvictimization—experiencing multiple types of victimization [2]—which can lead to emotional and behavioral issues, including substance use disorders [4]. In fact, the vast majority of justice-involved girls and women report multiple experiences of physical, sexual, and/or emotional abuse, and many report victimization as a key contributor to their offending [5,6,7]. Despite this robust association between victimization and female offending, many questions remain regarding influences of direct versus indirect victimization on offending [8,9,10,11,12]. The current study “unpacks” this relationship by examining how indirect and direct forms of victimization differentially affect girls’ delinquency outcomes, including fighting, substance abuse, running away, and sex work. We begin by reviewing existing knowledge of these two forms of victimization, their association with delinquent and criminal offending, and critical gaps in the research literature. We then examine independent and simultaneous influences of direct and indirect victimization, controlling for other factors, using data from 100 girls from juvenile justice facilities. We conclude by summarizing findings and discussing implications for health and public policy.

### 1.1. Theoretical Perspective & Definitions

Feminist-derived theories, such as the pathways perspective, focus on the role of victimization, along with other factors such as substance use and mental health problems, as important contributors to female-specific pathways to crime [13,14]. For instance, victimized girls may cope with the trauma by using substances or running away to escape the abuse; this may, in turn, cause them to turn to sex work or street crime to survive, as well as creating additional victimization risk [6]. Because most youths are in fact polyvictims [2], it is important to understand how different forms of victimization are related to negative outcomes.

Direct victimization is victimization that directly harms an individual [15,16,17]. Some examples include being assaulted, attacked with an object, or sexually abused. Indirect victimization, on the other hand, is victimization that an individual either is exposed to or has witnessed [17,18,19,20]. Examples of indirect victimization may include witnessing attacks, witnessing family violence, or knowing someone who was murdered. While studies have examined each in relationship to offending, it is possible that excluding one form in a particular study may misrepresent the impact of the other on the outcomes in question. For instance, if witnessing violence was excluded from a study, this might result in findings that appear to demonstrate a strong relationship of physical victimization with violent offending; yet that relationship may be overstated due to failure to account for variance attributable to witnessing violence—a common experience among polyvictims. Thus, attention to both forms of victimization is essential for researchers to garner a full picture of the victimization-offending link.

### 1.2. Links between Victimization & Offending

The link between victimization and offending is well established in the literature. For example, child sexual abuse has been identified as a strong predictor of violent and nonviolent criminal behavior among adolescents [21], and females in particular [9]. Many studies have found that both physical and sexual abuse leads to higher substance use [5,22,23,24]. Not surprisingly, girls who have a sexual abuse history are more likely to be arrested for drug offenses [25], and girls experiencing other forms of direct violent victimization (e.g., assault, attacked with/without a weapon) also report high levels of drug use [20,26].

There are also indications that indirect victimization, such as exposure to community violence or family violence, can lead to crime and delinquency. Indirect victimization, at home and in the community, has been found to increase both the likelihood and frequency of running away [27,28]. Further, exposure to domestic violence in the home has been linked with perpetrating physical and sexual assaults on minors [29]. Indirect victimization has been widely associated with substance use [11,30,31,32,33,34]. In fact, indirect victimization may be a better predictor of later substance use among females than direct victimization [20].

Yet, researchers have only recently begun to examine differential and cumulative effects of different types of victimization on offending. Fagan [15] found that experiencing both direct and indirect victimization is related to a higher frequency of offending compared to experiencing just one form of victimization in isolation. Similarly, exposure to multiple victimizations appears to increase the impact on later delinquency [35]. Pinchevsky and colleagues [20] reported that substance use increased with each additional form of violent victimization that a youth reported experiencing, while Acoca [5] found that having a history of multiple cases of sexual abuse can lead to a higher likelihood of using multiple drugs. There is also evidence that being repeatedly victimized increases the likelihood of repeat offending [36].

While there is strong evidence that experiencing a multitude of victimizations can lead to increased delinquent behavior, it is not easy to find distinct effects of direct or indirect forms of victimization. Because many studies have narrow measures of indirect and direct victimizations, as well as limited outcomes, a relationship between one type of victimization and delinquency outcome may be the effect of a separate, unmeasured variable, or even the result of an accumulation of variables that have not been analyzed [10]. Given the recent research on the differential effects of distinct forms of victimization as well as the effects of polyvictimization [17,26,35,37], Hahm and associates asserted that it is “no longer appropriate to assume that all types of maltreatment are equivalent in their potential contribution to developmental sequelae” [35] (p. 539). The current study adds to existing victimization and delinquency literature by examining the effects of indirect and direct victimization on delinquency outcomes for a sample of girls adjudicated delinquent.

## 2. Materials and Methods

### 2.1. Sampling

This study was reviewed and approved by a human-subjects institutional review board. Data used for this study was originally collected for a broader study of victimization and offending, and a full technical report describes procedures in detail [6]. Our sample included 100 girls, ages 12–18, who were adjudicated delinquent through a state department of juvenile justice. At the time of the study, the girls were housed at one of three facilities, as described by DeHart and Moran [7]. Prospective participants were exhaustively sampled from the system’s database, and each was invited to meet with the interviewer to learn more about the study. For those who assented (98%), the interview was conducted at that time, and girls received a $20 USD incentive in their spending accounts.

### 2.2. Measures

Archival data were obtained from the department of juvenile justice for participant demographics.

Life history calendars (LHC) [38] are used in interviews in order to capture an accurate picture of an interviewee’s life that allows a researcher to analyze the timing and sequencing of events in a respondent’s life [6]. These are especially useful for collecting accurate and sequential data, and also to help the respondent recall information that might have been forgotten. In this research, some common life events included schools, grades, living arrangements, and victimizations [6]. They provide a visual representation that usually arranges years across the top (i.e., ages, grades) and certain life events (i.e., victimization, offending) along the left-hand side of the page. The life events and age cues help youth recall details of their lives that might not be easily remembered [39,40]. While mapping events on the life calendars, the interviewer took field notes while talking to each youth and then transcribed their notes within 24 h.

Victimization measures on these LHCs were obtained from the Juvenile Victimization Questionnaire (JVQ) using a lifetime, retrospective technique [7,41]. The JVQ measures types of victimizations that are likely in both childhood and adulthood including child maltreatment, gang violence, dating violence, sexual victimization, and witnessing/indirect victimizations [41]. The validity of the JVQ is well established, including samples of youth [8].

Indirect victimization was constructed from seven variables that measured whether girls had ever experienced specific victimizations: Witnessed caregiver-on-caregiver violence, witnessed caregiver violence on children, witnessed attack with weapon, witnessed other bad attack without weapon, experienced having someone close murdered, witnessed murder or saw a dead body, and saw or heard shooting or rioting. Each victimization item was dichotomized indicating the presence (=1) or absence (=0) of each type of indirect victimization, and these were summed to create indirect victimization counts. Each girl could have a count ranging from 0–7 for this variable, indicating the number of different types of indirect victimization they had experienced. Direct victimization items, tallied similarly, included: Experienced attack with an object, experienced bad attack without object, experienced hate crime attack, experienced caregiver physical attack, experienced attack by gang or group, experienced attack by boyfriend or girlfriend, experienced sexual molestation by known adult, experienced sexual molestation by unknown adult, and experienced sexual assault by peer. Each girl could have a count ranging from 0–9 representing the number of types of direct victimization they experienced.

Offending history was obtained using girls’ self-reported involvement in substance use, fighting, running away, and sex work per the LHC. For purposes of the current analyses, Substance Use was a dichotomous variable indicating if they had ever used alcohol or drugs. Fighting represented whether or not the girl had ever engaged in fighting or hurting someone. Running Away represented having ever run away from home for a few days or longer. Sex work represented if a girl had ever engaged in sex work or traded sex for money, goods, or favors.

Control variables were chosen based on the risk factors which have been shown to be associated with delinquency, including demographic characteristics, family measures, and prior delinquency [18,42]. Age was measured as girls’ age at the time of the interview. Race was measured as a dichotomous variable (1 =white, 0= non-white). Three controls were used to measure the stability in each girls’ lives: Schools, Children in Home, and Adults in Home. “Schools” reflects the number of schools each girl attended and can indicate a transient lifestyle. Having a more unstable childhood is an indicator of delinquent behavior [43,44]. Children in Home was a measure indicating the total number of children living in each girl’s home. Victimization and delinquency are more common in large families [45,46]. Adults in Home is a measure of the total number of adults living in each girls’ home. Parent Crime is a dichotomous measure denoting whether the girl experienced a caregiver being sent to jail or prison. Parental involvement in crime could represent an absence of stability and lack of a stable home environment for the youth, and is a correlate of delinquent behavior [47]. Family Drinking/Drug Use is a dichotomous variable which measured whether each girl reported family drinking or drug problems. Finally, Prior Crime is a measure that specifically controls for the temporal ordering between a girl’s victimization and delinquency. It is a dichotomous measure indicating crime onset before victimization (coded as 1) rather than crime onset starting in the same year or after victimization (coded as 0).

### 2.3. Analyses

Logistic regressions were used to examine the associations between the two independent variables (Indirect Victimization, Direct Victimization) and the four dependent delinquency outcomes (Substance Use, Fighting, Running Away, Sex Work). First, tolerance values were checked to determine multicollinearity problems and no model reached 0.40 which would highlight a problem [48]. For each outcome, a series of regressions were performed. Model 1 includes the regression analysis for indirect victimization excluding control variables. Model 2 then adds the control variables. Model 3 examines the impact of direct victimization without controls, and Model 4 adds the controls. Finally, Model 5 includes both indirect and direct victimization along with the controls. Model 5, then, depicts the relative impact of indirect victimization, controlling for direct victimization with each delinquent outcome. After all analyses were completed, equality of coefficient tests were then performed to examine whether indirect and direct victimization elicited statistically different effects in the regression models. That is, indirect victimization coefficients in Model 1 were compared to direct victimization coefficients from Model 3, while indirect victimization coefficients in Models 2, which included control variables, were compared to direct victimization coefficients in Model 4, which also included the control variables [49].

## 3. Results

### 3.1. Descriptive Statistics

Table 1 provides the descriptive statistics for the sample. The girls, on average, were close to 16 years of age, and 65% were persons of color (e.g., black or Hispanic), while 35% were white. Girls had attended an average of 3.54 schools in their lifetime and had relatively few children in their homes (0.75 children on average). Only 37% of the youth had more than one adult in the home, indicating a large number of single parents or guardians. This could be explained by a high number of girls reporting a crime by a parent (54%) or a history of family drinking or drug problems (48%). This snapshot of the sample shows that many of the girls in the sample had a relatively unstable life characterized by changing schools, few adults or guardians in the home, and a presence of parental crime or substance use problems.

Before examining victimization histories and delinquency reported by each girl, it is important to note that 20% of the sample committed at least one crime prior to any victimization, but this is controlled for in the multivariate analyses. The sample had a high prevalence of delinquency, with 83% of girls indicating they used substances, 90% participating in fighting, 77% running away, and 14% engaging in sex work at some point in their lives. For indirect victimization, girls had experienced, on average, 2.97 out of the 7 types of victimization (counts). Girls experienced an average of 2.45 types of direct victimization out of 8. Frequencies of the raw data reveal that, overall, 85% of the girls experienced some type of direct victimization, and 91% experienced some type of indirect victimization.

### 3.2. Effects of Indirect and Direct Victimization on Substance Use

Table 2 examines the effects of indirect and direct victimization on substance use. As can be seen in the table, both indirect and direct victimization were related to substance use, although direct victimization decreased in significance (*p* ≤ 0.10) in models with controls (Models 4 and 5), while indirect victimization did not. Indirect victimization was positively related to substance use (*p* ≤ 0.05) (Model 1). This relationship was also significant when controls were added into the model (Model 2), and remained when direct victimization was included (Model 5). Direct victimization was related to substance use (*p* ≤ 0.05) with no controls included (Model 3), but decreased to marginal significance (*p* ≤ 0.10) once controls were added in Model 4. This trend remained in Model 5 when all controls and the effect of indirect victimization were included. Model 5 explains the most variation across all the models (39.5%). Equality of coefficients tests revealed no statistically significant differences in effects between indirect and direct victimization on substance use. In general, being white, attending more schools, and participating in prior crime before victimization were related to substance use in some of the models. White girls, as opposed to girls of color, were more likely to use substances across all models (this trend was marginal (*p* < 0.10) in Model 4). Girls who changed schools more often were also more likely to engage in substance use (Models 2 and 5). Those who committed crimes before victimization were slightly more likely (*p* ≤ 0.10) to engage in substance use when both indirect and direct victimization were included in Model 5.

### 3.3. Effects of Indirect and Direct Victimization on Fighting

Table 3 details the effects of indirect and direct victimization on fighting. Indirect and direct victimization were each related to fighting when controls were included (Models 2 and 4), but only direct victimization was significant by itself (*p* < 0.05, Model 3) and even increased in significance in Model 4 when controls were included. Direct victimization remained significant while controlling for indirect victimization (Model 5), suggesting that experiencing more forms of direct victimization increases the likelihood of fighting among girls. By including both indirect and direct victimization along with controls in Model 5, 49.9% of variance was explained—higher than any other model. Equality of coefficients tests, however, did not show any significant differences between indirect and direct victimization effects on fighting. The only other variable that was related to fighting was race: Across all models, white girls were less likely than girls of color to engage in fighting with others (although this was only a trend when indirect victimization was included in Model 2).

### 3.4. Effects of Indirect and Direct Victimization on Running Away

Table 4 assesses the effects of indirect and direct victimization on running away. The marginal (*p* < 0.10) effect of indirect victimization was limited only to Model 5 where direct victimization and controls were all entered into the model; by itself, indirect victimization was not related to the likelihood of running away (Model 1), nor was it significant in Model 2, when all control variables were added. Direct victimization was marginal (*p* ≤ 0.10) alone in Model 3 and remained when adding in controls (Model 4). Further, experiencing more forms of direct victimization increased the likelihood of running away (*p* ≤ 0.05) once indirect victimization was included (Model 5). No control variables were related to the likelihood of running away in any of the models. The variance explained was low in all models, but Model 5 explained the most variance in the outcome (18.7%).

Unlike the previous models related to substance use and fighting, there were notable findings in the equality of coefficient tests for running away. There was a trend (*p* ≤ 0.10) when comparing the effects of indirect and direct victimization in models with no control variables (Model 1 vs. Model 3). Additionally, there was a significant difference (*p* ≤ 0.05) between the impact of indirect and direct victimization on girls’ running away in models which included controls (Model 2 vs. Model 4). This pattern of results suggests that the impact of experiencing more varied forms of direct victimization has a stronger and positive impact on running away among girls than does experiencing more varied forms of indirect victimization. Additionally, because direct victimization was also significant in Models 3 and 4, as well as in Model 5 when indirect victimization was included, it appeared to have a stronger effect than indirect victimization on running away.

### 3.5. Effects of Indirect and Direct Victimization on Sex Work

Table 5 explores the effect of indirect and direct victimization on sex work. Indirect victimization was not related to sex work in any of the models. Direct victimization was related to sex work alone (Model 3) and when controlling for the effects of indirect victimization (Model 5). Generally, the models indicate that direct victimization is a stronger, and a statistically different, predictor of sex work than indirect victimization. Indirect victimization was not related to sex work alone (Model 1) or after adding in the effects of controls and direct victimization (Model 2, Model 5). Direct victimization alone (Model 3) was related to sex work (*p* ≤ 0.05), but this pattern was reduced (*p* ≤ 0.10) after controls were added into Model 4. By controlling for the effects of indirect victimization (Model 5), the number of types of direct victimization was again related to sex work (*p* ≤ 0.05). Including both indirect and direct victimization in Model 5 resulted in the most variation explained among the models (33.2%). There were a number of controls that were related to sex work, including age, family drinking and drug use, and the number of adults in the home. Across all models, older girls were more likely to engage in sex work (*p* ≤ 0.05). Girls who had a history of family drinking and drug use were slightly more likely (*p* ≤ 0.10) to engage in sex work with indirect victimization (Model 2) and with both indirect and direct victimization (Model 5). More adults in the home resulted in marginally decreasing the likelihood (*p* ≤ 0.10) of sex work in Model 4 with direct victimization, indicating that girls with fewer adults in the home would have an increased likelihood of participating in sex work.

Again, equality of coefficient tests exposed differences between indirect and direct victimization for sex work. There was a significant difference (*p* ≤ 0.05) when comparing indirect and direct victimization alone (Model 1 vs. Model 3). The significance level remained when comparing indirect and direct victimization in Model 2 and Model 4 with controls. These patterns indicate that the impact of experiencing various forms of direct victimization was stronger on sex work outcomes than experiencing indirect victimization. Because direct victimization was the only significant predictor in these models, this also suggests that a girls’ experience of forms of direct victimization has a stronger effect on sex work than indirect victimization.

## 4. Discussion

The current study examined the impact of both indirect and direct forms of victimization on substance use, violence, running away, and sex work among delinquent girls. Three major findings emerged. First, sex work and running away seem to be unique outcomes, as direct and indirect forms of victimization appear to operate in substantively different ways for these two types of delinquency. Direct victimization was a stronger predictor of a girls’ likelihood to run away and engage in sex work than was indirect victimization. Feminist theory may best explain how many of the girls become involved in these gendered crimes. Girls may use survival strategies [50,51,52] to escape their physical or sexual victimization, which leads them to run away. Furthermore, girls who run away may turn to sex work as an additional form of survival on the street [53,54]. Since the measure of direct victimization used in this study included physical abuse from a caregiver as well as sexual abuse, the findings support the notion that girls may run away and/or engage in sex work as a result of such victimization [24,28]. Thus, direct victimization appears to be an enormous threat to females, especially when it comes to running away and sex work outcomes.

The second main finding is that direct victimization appears to be a more salient predictor of delinquency, more so than indirect victimization, because direct victimization tended to maintain its effects even when controlling for indirect victimization. Trends in the data indicated that direct victimization was related to an increased likelihood of fighting, running away, sex work, and (marginally) substance use—even when controlling for the effect of indirect victimization. Thus, while both forms of victimization were related to substance use and running away, the impact of direct victimization was more consistent across outcomes. In particular, it may be that direct physical victimization has stronger links to behaviors such as aggression (e.g., stemming from fighting back against violence), running away (e.g., to escape physical violence), and survival sex (e.g., stemming from past sexual abuse). Yet, when one witnesses violence, including that at home or less frequent events like knowing someone who was murdered, the girl’s method of ‘escape’ may be to do so cognitively through numbing oneself with drugs and alcohol.

While the results of this study support prior research showing that direct victimization contributes to girls’ delinquency [9,55,56], the study is more variable regarding the impact of indirect victimization among girls. Prior evidence has suggested mixed findings for the effect of indirect victimization on violence [42,57], with some evidence that indirect victimization impacts substance use, running away, and sex work [20,24,27]. The current study found that indirect victimization increased the likelihood of substance use among girls but (marginally) decreased the likelihood of running away. Further, indirect victimization was not related to fighting or sex work when controlling for the impact of direct victimization. Taken together, the third and final major finding is that indirect victimization indeed appears to be linked to substance use among girls, but its effect on other delinquency outcomes remains somewhat mixed.

### Limitations & Directions for Future Research

The findings from this study enhance our current understanding regarding the effects of different forms (direct and indirect) of victimization on outcomes among delinquent girls, but there are limitations that future researchers might address. First, the study used retrospective self-reports. Thus, it may be helpful for studies to include triangulated administrative data, as well as prospective, longitudinal designs. Further, due to the retrospective, self-report design, it is possible that some forms of victimization were not captured during data collection. It is nearly impossible to gather information on all types of victimization experienced during a single study, and this may have impacted how either indirect or direct victimization was represented. For instance, perhaps indirect victimization is not as salient in recall and thereby not captured with the same degree of accuracy as direct victimization. This could, in turn, affect the strength and consistency of findings. Thus, researchers may wish to include cues specifically designed to elicit accuracy in reports of indirect and direct violence.

Another potential limitation is that indirect and direct victimization measures were not mutually exclusive, meaning, the girls could be (and often were) exposed to both direct and indirect forms of victimization. So, even if direct victimization mattered more for many of the outcomes, girls were likely to be victims of both forms of victimization simultaneously. Again, measures that more explicitly tease apart timing and effects of indirect versus direct exposure may be useful in improving future studies. Further, our dichotomization of victimization for purposes of using count data could oversimplify factors associated with degree or frequency of victimization; however, our own exploratory analyses indicate that alternate conceptualizations yielded nearly identical results to those presented here. Still, this remains a factor that could be explored in greater depth in future studies.

More generally, this study used data from a sample of delinquent girls from one state, which limits the generalizability of the findings to other populations. The study included no control group (e.g., non-delinquent girls) and no males, so opportunities for comparisons to these other groups are limited. Comparative research could demonstrate differential effects of victimization type on delinquency, informing theoretical perspectives on gendered pathways to offending. Future research could also examine the presence or absence of protective factors and how it relates to specific outcomes for youth.

## 5. Conclusions

The current study sheds light on serious impacts of child victimization, particularly in areas with substantial implications for public health. Direct victimization was linked to fighting, running away, and sex work—all of which create additional health and safety concerns. Fighting, of course, may be a precursor to more serious violent offending, while running away and sex work present substantial risks to those involved as well as risks to public health. Accordingly, it is imperative for our healthcare, human services, and justice systems to incorporate a strengthened understanding of risks and needs for delinquent youth. Understanding the role of victimization, including the relative impacts of different forms of victimization, can help inform practice and policy. For instance, understanding the importance of indirect victimization on subsequent substance abuse can inform prevention, risk reduction, and treatment programs for youth—particularly those who may face compounded risk in being homeless or transitional. Similarly, the severity of risks represented by direct victimization for behaviors such as running away and sex work necessitates focused approaches to ameliorate impacts of victimization. This relationship also serves as an important point of information in addressing the criminalization of survival strategies.

It is imperative for all justice systems, from the police to detention facilities, to have a better understanding of risks and needs for youth, particularly females, who are delinquent. Understanding their full victimization history (types, counts, and frequencies) is necessary in order to address the needs of the youth and hopefully put them in programs that will aid their successful reentry back to the community. It is also important for those employed in the criminal justice system to understand girls’ protective factors, or what their strengths are that would help them succeed.

Prevention programs for youth can help to provide pro-social support increasing protective and resiliency factors that may help to curb delinquency or future recidivism among youth. Since this study found direct victimization was more important than indirect victimization, prevention efforts should specifically target direct victimization among girls. Programs should also address post-victimization issues that may lead to delinquency. Because there is a strong relationship between direct victimization and running away and sex work, efforts should focus on these areas, although programs may also address direct victimization risks associated with substance use and fighting as well. These intervention efforts should be directed at middle school and high school aged youth, as this is a time of particular risk for girls to turn towards illegal activities and hanging around delinquent peers. Interventions for younger children should be directed toward problems at home, such as family violence or abuse.

Policymakers must attend to the very real threats in the lives of youth rather than adding to the trauma in their lives via criminalization or punitive systemic responses. Understanding the role that victimization and other traumas play in delinquent youths’ lives is essential to address unmet needs, identify solutions that do not involve incarceration, and prepare detained youth for successful reentry back into communities.

## Figures and Tables

**Table 1:** Descriptive statistics.

Variables	Mean/%	SD	Min–Max
**Dependent Variables**			
Substance Use	0.83	0.38	0–1
Fighting	0.90	0.30	0–1
Running Away	0.77	0.42	0–1
Sex work	0.14	0.35	0–1
**Independent Variables**			
INDIRECT Counts	2.97	1.80	0–7
DIRECT Counts	2.45	1.79	0–8
**Control Variables**			
Age	15.67	1.17	12–18
White	0.35	0.48	0–1
Schools	3.54	2.18	0–7
Children in Home	0.75	0.90	0–4
Adults in Home	0.37	0.49	0–1
Parent Crime	0.54	0.50	0–1
Family Drinking/Drug Use	0.48	0.50	0–1
Prior Crime	0.20	0.40	0–1

**Table 2:** The effect of indirect and direct victimization counts on substance use.

Substance Use	Model 1	Model 2	Model 3	Model 4	Model 5
**Independent Variables**					
INDIRECT Counts	0.398 * (0.174)	0.567 * (0.246)	—	—	0.544 * (0.258)
DIRECT Counts	—	—	0.537 * (0.210)	0.481 ^†^ (0.252)	0.446 ^†^ (0.261)
**Controls**					
Age	—	0.073 (0.268)	—	0.034 (0.270)	−0.077 (0.288)
White	—	1.938 * (0.867)	—	1.443 ^†^ (0.855)	1.951 * (0.934)
Schools	—	0.337 ^†^ (0.181)	—	0.269 (0.169)	0.322 ^†^ (0.183)
Parent Crime	—	−0.136 (0.674)	—	−0.259 (0.646)	−0.344 (0.709)
Family Drinking/Drug Use	—	−0.060 (0.722)	—	0.122 (0.680)	−0.273 (0.748)
Prior Crime	—	1.434 (0.962)	—	1.314 (0.915)	1.949 ^†^ (1.030)
Children in Home	—	0.427 (0.513)	—	0.388 (0.455)	0.488 (0.486)
Adults in Home	—	−0.256 (0.818)	—	0.240 (0.752)	−0.117 (0.837)
Constant	0.590 (0.475)	−2.834 (4.073)	0.597 (0.432)	−1.513 (4.058)	−1.226 (4.248)
Nagelkerke	0.100	0.348	0.137	0.305	0.395

^†^*p* ≤ 0.10; * *p* ≤ 0.05; ** *p* ≤ 0.01; *** *p* ≤ 0.001.

**Table 3:** The effect of indirect and direct victimization counts on fighting.

Fighting	Model 1	Model 2	Model 3	Model 4	Model 5
**Independent Variables**					
INDIRECT Counts	0.282 (0.201)	0.740 * (0.375)	—	—	0.170 (0.462)
DIRECT Counts	—	—	0.692 * (0.285)	1.739 ** (0.621)	1.615 * (0.649)
**Controls**					
Age	—	−0.289 (0.379)	—	−0.504 (0.549)	−0.503 (0.548)
White	—	−1.493 ^†^ (0.906)	—	−3.460 ** (1.349)	−3.143 * (1.441)
Schools	—	0.164 (0.190)	—	0.050 (0.234)	0.097 (0.252)
Parent Crime	—	0.067 (0.951)	—	0.334 (0.975)	0.264 (1.063)
Family Drinking/Drug Use	—	−0.931 (0.910)	—	−0.995 (1.033)	−1.128 (1.137)
Prior Crime	—	0.084 (1.074)	—	−0.146 (1.163)	0.133 (1.376)
Children in Home	—	0.309 (0.610)	—	0.363 (0.626)	0.334 (0.616)
Adults in Home	—	−1.241 (0.942)	—	−1.420 (1.121)	−1.354 (1.097)
Constant	1.412 * (0.571)	5.946 (5.890)	0.971 * (0.493)	9.722 (8.487)	9.171 (8.417)
Nagelkerke	0.045	0.286	0.163	0.494	0.499

^†^*p* ≤ 0.10; * *p* ≤ 0.05; ** *p* ≤ 0.01; *** *p* ≤ 0.001.

**Table 4:** The effect of indirect and direct victimization counts on running away.

Running Away	Model 1	Model 2	Model 3	Model 4	Model 5
**Independent Variables**					
INDIRECT Counts	**−0.102** (0.134)	***−0.149*** (0.159)	—	—	−0.395 ^†^ (0.204)
DIRECT Counts	—	—	**0.271**^†^ (0.153)	***0.357***^†^ (0.185)	0.505 * (0.204)
**Controls**					
Age	—	0.043 (0.226)	—	−0.097 (0.226)	0.020 (0.242)
White	—	0.355 (0.561)	—	0.188 (0.580)	−0.205 (0.639)
Schools	—	0.048 (0.125)	—	−0.007 (0.121)	0.013 (0.128)
Parent Crime	—	−0.208 (0.530)	—	−0.586 (0.546)	−0.328 (0.569)
Family Drinking/Drug Use	—	0.364 (0.529)	—	0.128 (0.539)	0.473 (0.570)
Prior Crime	—	0.150 (0.672)	—	0.558 (0.679)	0.188 (0.704)
Children in Home	—	−0.482 (0.327)	—	−0.406 (0.328)	−0.501 (0.337)
Adults in Home	—	0.981 (0.654)	—	0.844 (0.635)	0.952 (0.655)
Constant	1.466 ** (0.484)	0.579 (3.434)	0.591 (0.393)	2.048 (3.442)	0.890 (3.622)
Nagelkerke	0.009	0.076	0.052	0.124	0.187

^†^*p* ≤ 0.10; * *p* ≤ 0.05; ** *p* ≤ 0.01; *** *p* ≤ 0.001; Note: Bolded coefficients indicate significant differences in magnitude of effects (*p* ≤ 0.10) in models 2 vs. 4 and in models 1 vs. 3. Bolded and italicized coefficients indicate significant differences at the *p* ≤ 0.05 level.

**Table 5:** The effect of indirect and direct victimization counts on sex work.

Sex Work	Model 1	Model 2	Model 3	Model 4	Model 5
**Independent Variables**					
INDIRECT Counts	**−0.041** (0.162)	**−0.163** (0.210)	—	—	−0.384 (0.243)
DIRECT Counts	—	—	**0.331** * (0.158)	**0.380**^†^ (0.204)	0.524 * (0.228)
**Controls**					
Age	—	0.816 * (0.364)	—	0.813 * (0.384)	0.838 * (0.389)
White	—	−0.137 (0.663)	—	−0.250 (0.680)	−0.295 (0.691)
Schools	—	−0.069 (0.161)	—	−0.089 (0.173)	−0.077 (0.176)
Parent Crime	—	0.721 (0.675)	—	0.479 (0.692)	0.522 (0.721)
Family Drinking/Drug Use	—	1.455 ^†^ (0.758)	—	1.106 (0.717)	1.441 ^†^ (0.783)
Children in Home	—	−0.567 (0.512)	—	−0.510 (0.519)	−0.414 (0.546)
Adults in Home	—	−1.440 (0.924)	—	−1.746 ^†^ (0.980)	−1.593 (1.018)
Constant	−1.649 ** (0.548)	−14.383 * (5.793)	−2.733 *** (0.586)	−15.483 * (6.225)	−15.452 * (6.248)
Nagelkerke	0.001	0.241	0.079	0.292	0.332

^†^*p* ≤ 0.10; * *p* ≤ 0.05; ** *p* ≤ 0.01; *** *p* ≤ 0.001; Note: Bolded coefficients indicate significant differences in magnitude of effects (p≤ 0.10) in models 2 vs. 4 and in models 1 vs. 3.
